# Burden of respiratory syncytial virus in older adults in Brazil: insights from national surveillance data for the 2022-2023 period

**DOI:** 10.36416/1806-3756/e20250068

**Published:** 2025-07-22

**Authors:** Ana L Bierrenbach, Olivia T Ranzani

**Affiliations:** 1. Precision Data, São Paulo (SP) Brasil.; 2. Instituto de Ensino e Pesquisa, Hospital Sírio-Libanês, São Paulo (SP) Brasil.; 3. Santa Casa de Misericórdia de Mogi Mirim, Mogi Mirim (SP) Brasil.; 4. Olita Pesquisas Científicas Ltda, Mogi Mirim, São Paulo (SP) Brasil.

**Keywords:** Respiratory syncytial virus infections, Severe acute respiratory syndrome, Hospitalization, Epidemiology, Aged, Surveillance

## Abstract

**Objective:**

Respiratory syncytial virus (RSV) is a major cause of severe respiratory infections in older adults, particularly those with comorbidities. Despite its clinical impact, RSV remains underdiagnosed and underreported. We sought to assess the burden of RSV in older adults (≥ 60 years of age) in Brazil using national surveillance data for the 2022-2023 period.

**Methods:**

We analyzed RSV cases reported in the Sistema de Informação de Vigilância Epidemiológica, identifying them among reported cases of SARS. Cases were examined by demographic characteristics, seasonal trends, and clinical outcomes. RSV cases were compared across defined etiologies.

**Results:**

Among 355,230 reported cases of SARS in older adults, 201,965 (56.8%) had a defined etiology, and 1,465 (0.7%) were confirmed as RSV cases. Cases peaked in the second quarter of each year, with the highest incidence in the southern and southeastern regions. Despite a low hospitalization rate (2.3 per 100,000 population), severe outcomes were common: 30.4% required ICU admission, and 24.9% resulted in death, with mortality being highest in those ≥ 90 years of age.

**Conclusions:**

RSV-related hospitalizations in Brazil appear underestimated, with reported cases likely representing the most severe spectrum due to underreporting and diagnostic limitations. Seasonal patterns peaked in April-May, and regional differences highlight a higher incidence in the southern and southeastern regions, likely due to epidemiological factors and diagnostic disparities. Although the recent approval of RSV vaccines offers an opportunity to reduce disease burden, successful implementation requires broader access and inclusion in the Brazilian National Immunization Program. Strengthening surveillance, diagnostic capacity, and reporting processes is critical for better disease assessment and public health planning.

## INTRODUCTION

Respiratory syncytial virus (RSV) is a major cause of respiratory infections in older adults (≥ 60 years of age), particularly those with preexisting health conditions such as cardiopulmonary disease, immunosuppression, and frailty.^1^ Individuals with chronic conditions such as COPD, heart failure, and diabetes are at an increased risk of severe outcomes.^2^ ^,^ ^3^ Immunocompromised individuals, including those undergoing solid organ transplantation and receiving immunosuppressive therapy, are also highly susceptible.^4^ Older adults may contract RSV through close contact with children in their households or exposure in long-term care facilities and health care settings.^5^

Severe RSV infections in older adults often lead to complications such as pneumonia and exacerbations of underlying conditions, resulting in increased hospitalizations and mortality.^6^ ^,^ ^7^ Studies have shown that RSV is a significant contributor to respiratory hospitalizations in this age group, second only to influenza among viral causes of cardiopulmonary admissions. Despite its impact, RSV remains underdiagnosed and underreported in older adults due to the nonspecific nature of symptoms, limited awareness among health care providers, and challenges in diagnostic testing availability and utilization.^7^ ^-^ ^10^ In many cases, RSV infections may be misattributed to other respiratory pathogens, such as influenza or bacterial infections, further complicating public health assessments. 

In developed countries, RSV incidence rates in older adults have been estimated at approximately 600 cases per 100,000 person-years, with hospitalization rates reaching 157 per 100,000 person-years.^7^ ^,^ ^11^ ^,^ ^12^ In Latin America, systematic reviews have shown that older adults, particularly those > 65 years of age, face a high incidence of severe RSV-related outcomes, including ICU admissions and increased lethality.^13^ RSV is also a significant cause of respiratory infections in this population, contributing to hospitalizations for influenza-like illness and pneumonia.^14^ Despite these findings, data gaps persist, highlighting the need for further research to guide prevention and management strategies.^15^

In Brazil, RSV vaccines have recently been approved for older adults and pregnant women, representing an important step toward protecting these vulnerable populations. Additionally, vaccinating pregnant women and eventually infants may provide indirect protection for older adults by reducing virus circulation within the community. However, because these vaccines have yet to be incorporated into the Brazilian *Programa Nacional de Imunização* (PNI, National Immunization Program), access is still limited for those at an increased risk. Nevertheless, passive immunization with monoclonal antibodies has been incorporated into the PNI for certain high-risk pediatric populations, and additional antibody therapies are currently being developed and are expected to be available in Brazil soon. 

The objective of the present study was to provide a comprehensive description of the burden of RSV among older adults (≥ 60 years of age) in Brazil, using data from the *Sistema de Informação de Vigilância Epidemiológica* (SIVEP, Information System for Epidemiological Surveillance) for the 2022-2023 period (i.e., the post-COVID-19 period). Our analysis focused on demographic and seasonal patterns, as well as the proportion of RSV cases among reported cases of SARS and those with confirmed etiology. 

## METHODS

### Data source

The SIVEP was implemented in 2000 as a sentinel surveillance system for flu-like syndromes. During the 2009 influenza A (H1N1) pandemic, the SIVEP was expanded to include SARS cases, the reporting of which became mandatory nationwide.^16^ In 2020, the SIVEP was further modified to include COVID-19 cases. All hospitalized SARS cases and related deaths must be reported within 24 h by all registered health care facilities, regardless of hospitalization status. 

The definition of flu-like syndrome requires at least two symptoms, such as fever, chills, sore throat, headache, cough, and loss of taste/smell. Severe cases, classified as SARS cases, involve respiratory distress, persistent chest pain, oxygen saturation below 95%, or cyanosis. Hospitalized flu-like cases that do not meet the criteria for SARS must be reported in the Brazilian Unified Health Care System reporting system *e-SUS Notifica* instead. All public and private health care facilities must report SARS cases, and epidemiological surveillance personnel at each facility are responsible for completing reporting forms in accordance with Brazilian National Ministry of Health guidelines.^17^ ^,^ ^18^

The SIVEP database is publicly available on the Brazilian Unified Health Care System Information Technology Department website, ensuring patient confidentiality as it contains no identifying information. Therefore, in accordance with Brazilian regulations, ethical approval was not required for the present study. 

### Case definitions

The classification of SARS cases in the dataset was based on the CLASSI_FIN variable, which includes five distinct categories: (1) “SARS by influenza,” (2) “SARS by another respiratory virus,” (3) “SARS by another specified agent,” (4) “SARS unspecified,” and (5) “SARS by COVID-19.” COVID-19 cases were identified by using the classification “SARS by COVID-19,” whereas influenza cases were defined by the classification “SARS by influenza.” RSV cases were identified within the “SARS by another respiratory virus” category and required additional confirmation through a positive result in either the rapid antigen test (AN_VSR) or the PCR test (PCR_VSR) variables. Cases classified as “SARS by another respiratory virus” but without a confirmed RSV test result, along with those classified as “SARS by another specified agent,” were categorized under “Other defined etiology.” Finally, cases classified as “SARS unspecified” were grouped under the category of “Other undefined etiology.” 

All reported cases were considered to be SARS cases following the surveillance definition. We also analyzed a subdivision of SARS cases with a defined etiology, which included all categories except “Other undefined etiology.” 

### Analysis

Cases were analyzed by sex, age group (60-69, 70-79, 80-89, and ≥ 90 years), hospitalization status (hospitalized vs. non-hospitalized), ICU admission (ICU vs. non-ICU), Brazilian macroregion (central-west, northeastern, northern, southeastern, and southern), year, quarter, and risk factors. The database considered individual conditions such as chronic cardiovascular, hematologic, liver, neurological, pulmonary, and kidney diseases; Down syndrome; asthma; diabetes mellitus; immunodeficiency/immunosuppression; obesity; and other specified conditions. Cases were also classified by treatment in public or private facilities by using the Brazilian National Registry of Health Care Facilities, which compiles data on health care facilities in Brazil and was deterministically linked to the SIVEP for this analysis. Results were presented in frequency tables and graphs to highlight temporal and demographic trends. 

Reporting rates were calculated by year, sex, age group, and region by using 2022 Brazilian Institute of Geography and Statistics population estimates and were expressed per 100,000 population. Two RSV case proportions were determined: RSV-positive cases among all SARS reports and RSV-positive cases among SARS cases with a defined etiology. These proportions were further stratified by all analyzed variables. Differences were assessed by chi-square tests. 

Data management, analysis, and deterministic linkage were conducted with Stata software, version 17 (StataCorp LLC, College Station, TX, USA). 

## RESULTS

During the study period, 835,234 SARS cases were reported across all age groups, of which 401,107 (48.0%) had a defined etiology and 44,731 (11.2%) were confirmed as RSV cases. Among older adults (≥ 60 years of age), 355,230 cases accounted for 42.5% of the total number of SARS cases, with 201,965 (56.8%) having a defined etiology and 1,465 (0.7%) being confirmed as RSV cases. 

Table 1 shows that COVID-19 accounted for the largest proportion of SARS cases (52.4%), with the highest proportion in the first quarter of 2022 (63.1%) and the lowest in the second quarter of 2023 (25.2%). RSV represented 0.4% of all cases, peaking at 1.5% in the second quarter of 2023 and reaching its lowest proportion of 0.1% in the fourth quarters of both years. Influenza contributed to 2.4% of SARS cases, with notable peaks in the first quarter of 2022 (2.7%) and the second quarter of 2023 (9.3%). Other defined etiologies accounted for 6.3% of cases, with the highest proportion observed in the third quarter of 2023 (9.5%). Cases with undefined etiologies made up 38.5% of the total, varying from 29.2% in the first quarter of 2022 to 60.9% in the third quarter of 2023. 

**Table 1:** Quarterly distribution of SARS cases among older adults (≥ 60 years of age) in Brazil for the 2022-2023 period, by etiology.^a^

Etiology	2022				2023				Total
	Quarter 1	Quarter 2	Quarter 3	Quarter 4	Quarter 1	Quarter 2	Quarter 3	Quarter 4	
COVID-19	81,313 (63.1)	29,884 (51.3)	19,613 (47.4)	24,775 (59.4)	10,948 (44.1)	6,002 (25.2)	4,735 (27.4)	8,938 (46.6)	186,208 (52.4)
RSV	263 (0.2)	468 (0.8)	53 (0.1)	43 (0.1)	146 (0.6)	361 (1.5)	109 (0.6)	22 (0.1)	1,465 (0.4)
Influenza	3,461 (2.7)	725 (1.2)	590 (1.4)	593 (1.4)	436 (1.8)	2,221 (9.3)	276 (1.6)	141 (0.7)	8,443 (2.4)
Other defined	6,130 (4.8)	3,571 (6.1)	2,798 (6.8)	3,193 (7.7)	1,654 (6.7)	1,885 (7.9)	1,641 (9.5)	1,487 (7.8)	22,359 (6.3)
Other undefined	37,600 (29.2)	23,613 (40.5)	18,327 (44.3)	13,122 (31.4)	11,628 (46.9)	13,384 (56.1)	10,509 (60.9)	8,572 (44.7)	136,755 (38.5)
Total	128,767 (100.0)	58,261 (100.0)	41,381 (100.0)	41,726 (100.0)	24,812 (100.0)	23,853 (100.0)	17,270 (100.0)	19,160 (100.0)	355,230 (100.0)

RSV: respiratory syncytial virus. ^a^Values expressed as n(%).

**Figure 1:**
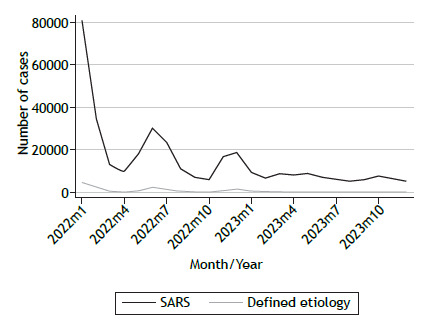
Seasonal distribution of all reported cases of SARS and those with defined etiology among older adults (≥ 60 years of age) in Brazil for the 2022-2023 period.

Figure 1 illustrates the seasonal distribution of all reported cases of SARS among older adults, including those with a defined etiology. Throughout the study period, the trend of SARS cases with a defined etiology closely mirrors that of total SARS cases, running almost parallel and indicating that a substantial proportion of reported cases had a confirmed cause. The prominent peak observed in early 2022 corresponds to the end of the third COVID-19 wave in Brazil. 

**Figure 2:**
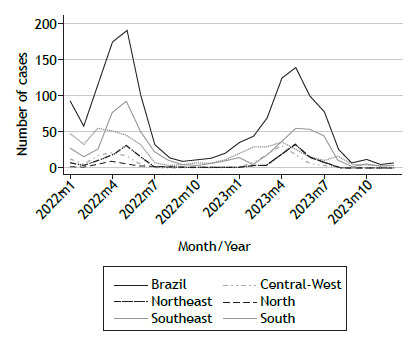
Seasonal distribution of laboratory-confirmed respiratory syncytial virus cases among older adults (≥ 60 years of age) across the five regions of Brazil for the 2022-2023 period.

Figure 2 illustrates the seasonal distribution of laboratory-confirmed RSV cases reported among older adults (≥ 60 years of age) across the five regions of Brazil, showing peaks generally occurring around April and May, with some regional variations. The overall number of RSV cases is considerably lower in comparison with the total number of SARS cases shown in Figure 1, highlighting the contribution of various respiratory pathogens to the burden of severe infections in this population. The southeastern region of Brazil consistently reported the highest number of cases, followed by the southern region, whereas the northern region recorded the lowest numbers. These regional differences may be influenced by variations in laboratory capacity and reporting practices. 

Table 2 presents the distribution of confirmed RSV cases stratified by various demographic and clinical characteristics. Of the 1,465 reported cases, a higher proportion occurred in 2022 (56.5%) in comparison with 2023 (43.5%). The proportion of cases among total SARS cases and cases with a defined etiology was higher in 2023 than in 2022. Women accounted for a larger share of cases (59.7%) than did men (40.3%). The highest proportion of cases occurred among those in the 70- to 79-year age bracket (33.6%). Rates increased with age. Hospitalization was common, with 96.1% of reported cases requiring hospital admission and 30.4% requiring intensive care. Mortality was 24.9%, increasing with age: 15.7% among individuals in the 60- to 69-year age bracket; 24.6% among those in the 70- to 79-year age bracket; 30.3% among those in the 80- to 89-year age bracket; and 43.6% among those ≥ 90 years of age. Most cases (82.5%) had underlying risk factors. Seasonal distribution shows that the majority of cases occurred in the second quarter of each year, with the highest proportion consistently in this period and the lowest in the fourth quarter. Regarding geographic distribution, the southern and southeastern regions accounted for the highest shares, followed by the central-west and northern regions. The highest rate was observed in the southern region, followed by the central-west and northern regions. RSV cases were nearly evenly distributed between public (46.6%) and private (53.4%) health care facilities. The values of p indicate that most comparisons were statistically significant, with the majority below 0.001 and some above this threshold but still significant at p < 0.05. However, age group (p = 0.085 for RSV+/SARS) and health care sector distribution (p = 0.763 for RSV+/SARS) did not reach statistical significance, whereas health care sector distribution (p = 0.002 for RSV+/Defined etiology) remained significant at p < 0.05. 

**Table 2:** Distribution and rates of confirmed respiratory syncytial virus cases among older adults (≥ 60 years of age) in Brazil for the 2022-2023 period, by demographic and clinical characteristics.

Category	N	Category/RSV+, %	Rate per 100,000	% RSV+/SARS	% RSV+/Defined etiology
Total	1,465		2.3	0.41	0.67
2022	827	56.5	2.6	0.31	0.5
2023	638	43.5	1.9	0.75	1.71
Female	874	59.7	2.4	0.47	0.85
Male	591	40.3	2.1	0.35	0.59
60-69 years	401	27.4	1.1	0.44	0.84
70-79 years	492	33.6	2.5	0.43	0.78
80-89 years	416	28.4	5.5	0.39	0.65
≥ 90 years	156	10.7	11.3	0.37	0.59
Quarter 1 (q1)	409	27.9		0.27	0.42
Quarter 2 (q2)	829	56.6		1.01	2.00
Quarter 3 (q3)	161	11.1		0.28	0.6
Quarter 4 (q4)	65	4.4		0.11	0.18
q1_2022	263	31.8		0.2	0.31
q2_2022	468	56.6		0.8	1.46
q3_2022	53	6.4		0.13	0.25
q4_2022	43	5.2		0.1	0.17
q1_2023	146	22.9		0.59	1.22
q2_2023	361	56.6		1.51	3.81
q3_2023	109	17.1		0.63	1.76
q4_2023	22	3.4		0.11	0.23
Central-West	156	10.9	3.6	0.58	0.90
Northeast	166	11.3	1.0	0.29	0.58
North	64	4.4	1.8	0.47	0.84
Southeast	491	33.5	1.6	0.27	0.46
South	585	39.9	5.5	0.80	1.43
Hospital	1,408	96.1		0.42	0.73
Non-hospital	39	2.7		0.53	0.92
Unknown	18	1.2		0.18	0.46
ICU	446	30.4		0.42	0.69
Non-ICU	890	60.8		0.46	0.79
Unknown/Non-hospital	129	8.8		0.24	0.52
Dead	364	24.9		0.35	0.54
Alive	1,101	75.1		0.44	0.82
Risk factor +	1,208	82.5		0.45	0.76
Risk factor −	257	17.5		0.30	0.59
Public facility	682	46.6		0.42	0.73
Private facility	783	53.4		0.41	0.62

RSV: respiratory syncytial virus.

## DISCUSSION

The present study analyzed the burden of RSV in older adults (≥ 60 years of age) in post-COVID-19 Brazil on the basis of national surveillance data for the 2022-2023 period. Our findings show a low hospitalization incidence (2.3 per 100,000 population), a low RSV proportion among SARS cases (0.41%), and high rates of underlying conditions, ICU admissions, and mortality. Nearly one third of hospitalized cases required intensive care, and approximately 25% died. These results highlight the need for targeted prevention and management, considering potential underreporting of milder cases. 

During the COVID-19 pandemic, Brazil expanded PCR testing to SARS-CoV-2, with mass testing in some areas and coverage by health insurance plans.^19^ However, access to other viral PCR tests varies by resource availability and regional policy.^20^ ^,^ ^21^ Public health care facilities offer these tests free of charge, whereas private facilities may charge for them, limiting access for uninsured individuals and even some insured, depending on the coverage. Although PCR testing for COVID-19 for those who have private health insurance is mandated by the Brazilian National Health Insurance Agency, coverage of other molecular tests depends on individual plan conditions and regulations. 

The reporting of SARS cases is compulsory in Brazil, and individuals hospitalized for acute lower respiratory infection are most commonly classified as being SARS cases.^18^ Non-hospitalized cases resulting in death and cases of patients treated exclusively in emergency rooms-often for extended periods while awaiting hospital beds-must also be reported to the SIVEP. Patients testing negative or untested should still be reported under SARS criteria. Assessing potential bias toward more severe cases in the SIVEP is crucial. Our findings suggest that such a bias occurred, as shown by our research team in a study that is currently under review and that linked SIVEP records with 2022-2023 Brazilian Unified Health Care System Hospital Information System records. Notably, 85% of ICD-10-coded RSV records in the Hospital Information System had not been reported in the SIVEP, despite the fact that reporting is mandatory. These results indicate significant underreporting in Brazil, with reported cases likely representing the most severe spectrum. This bias likely explains the low hospitalization incidence, low RSV proportion among SARS cases, and high ICU admission and mortality rates observed in our study. 

This context suggests that access to diagnostic tests plays a major role in observed differences in RSV incidence. While the incidence rate of RSV hospitalizations in our study was low at 2.3 per 100,000 population-comparable to low-resource settings and significantly lower than the 190-254 per 100,000 population reported in the USA-this disparity may be at least partially influenced by differences in health care access, alongside possible true epidemiological variations.^22^ ^,^ ^23^ The proportion of RSV cases among the total number of SARS cases was also low (0.41%), with a slight increase from 0.31% in 2022 to 0.75% in 2023. A meta-analysis found that among all patients with acute respiratory infection, RSV accounted for 1-10% in adults and 2-14% in patients with chronic diseases or transplant recipients, most of whom were hospitalized.^23^ Although testing capacity improved during the COVID-19 pandemic, timely access to specific tests in both public and private health care settings remains a critical factor in surveillance and disease burden assessment. 

Underreporting and underascertainment of RSV cases are well-documented challenges in surveillance systems. Studies indicate that RSV hospitalization rates are often underestimated because of limited diagnostic testing and reporting. In a systematic review and modeling study adjusted for diagnostic underascertainment, hospitalization rates among older adults were shown to be approximately 2.2 times higher than previously reported.^7^ This underascertainment may be especially pronounced for RSV in older adults, given that RSV is still widely perceived as a disease that primarily affects children. As a result, clinical suspicion for RSV in elderly patients tends to be low, and diagnostic testing is often not pursued by physicians or geriatricians.^24^ While other respiratory infections such as influenza and COVID-19 are also subject to underreporting and data limitations, RSV in older adults is particularly affected by diagnostic neglect, contributing to a more significant severity bias in reported cases. The implications of severity bias in reported cases, particularly in older adults, are further explored later in the discussion. These findings highlight the need to enhance surveillance strategies to capture the true burden of RSV and inform public health interventions. 

Despite a decline in reported SARS cases from 2022 to 2023, the proportion of RSV-positive cases among those with a defined etiology increased. This decline likely reflects the absence of a significant COVID-19 wave in 2023, whereas higher numbers in 2022 correspond to the end of the Omicron wave.^25^ Notably, the first RSV peak coincided with a peak in total SARS cases, suggesting RSV contributed modestly to the overall SARS burden. 

The seasonal peaks of RSV cases in April and May coincide with autumn in Brazil. This pattern aligns with findings from other regions, where RSV tends to peak earlier than influenza, often in late autumn or early winter.^26^ ^,^ ^27^ In 2022, the first year of the study, the RSV peak was followed by a SARS peak in June, suggesting a slightly earlier RSV season in comparison with other respiratory viruses. However, this pattern was not observed in 2023. Regional variations in peak timing could also be influenced by climate differences, such as rainfall patterns. While RSV seasonality in southern Brazil generally coincides with colder months, in central and northern regions it aligns more closely with the rainy season.^28^ ^,^ ^29^ Although some variation in peak timing was observed across regions, the overall seasonal pattern remains relatively similar. Additionally, differences in health care access, health care-seeking behavior, diagnostic capacity, and reporting practices across regions may further contribute to these variations. 

Geographic analysis showed the highest RSV case numbers and incidence rates in the southern and southeastern regions of Brazil, where colder temperatures may increase transmission. These regions also have better health care infrastructure and laboratory capacity, with detection and reporting therefore being better.^30^ However, pronounced seasonality suggests that the higher incidence reflects true epidemiological patterns rather than differences in health care access. Lower detection in the northern and northeastern regions may result from underdiagnosis and a genuinely lower incidence. 

Clinically, a significant proportion of RSV cases required hospitalization, with nearly one third requiring intensive care and approximately 25% resulting in death. In developed countries, in-hospital case-fatality rates for older adults with RSV range from 1.6% to 7.1%,^7^ ^,^ ^8^ ^,^ ^31^ although a systematic review reported an in-hospital case-fatality rate of 11.0% in adults with comorbidities^.^ ^3^ Despite the high comorbidity prevalence in our study, the nearly 25% fatality rate is markedly higher, suggesting disparities in health care access, disease severity, or population characteristics. This discrepancy likely reflects reporting bias, with more severe cases being captured in the surveillance system. 

Older adults in the 70- to 79-year age bracket accounted for the largest share of RSV cases, whereas those ≥ 90 years of age had the highest incidence and mortality rates, reflecting their greater vulnerability. This aligns with global findings that advanced age is a key risk factor for severe RSV.^12^ ^,^ ^22^ Women made up a higher proportion of cases, although no clear sex-based difference in RSV incidence is established. This disparity may stem from health care utilization patterns or underlying conditions, warranting further study. Most cases involved individuals with comorbidities, reinforcing the need for targeted interventions in high-risk populations.^23^ ^,^ ^32^

The recent approval of RSV vaccines for older adults in Brazil marks a key step in reducing disease burden in this high-risk group. These vaccines provide direct protection and may lower transmission when combined with maternal and infant vaccination programs. However, their impact depends on broad accessibility and inclusion in the PNI, which is still pending. Equitable access through public health programs is crucial, especially for vulnerable populations. As seen in other countries, robust surveillance and ongoing epidemiological assessments are essential to guide vaccination policies, optimize coverage, and evaluate real-world effectiveness. Strengthening these efforts will help reduce RSV-related morbidity and mortality in older adults. 

This study highlights the burden of RSV among older adults in Brazil, revealing key seasonal, demographic, and regional patterns. Findings reinforce the significant impact of RSV and the urgent need for targeted interventions and improved surveillance. Regional and seasonal variations emphasize tailoring prevention strategies and resource allocation to local epidemiology. Addressing diagnostic gaps and improving reporting processes are critical for accurate disease assessment. Integrating RSV vaccines into national immunization strategies could significantly reduce morbidity and mortality. Continued monitoring and research are essential for evidence-based public health policies that effectively meet the needs of older adults in Brazil. 
